# Materials descriptors of machine learning to boost development of lithium-ion batteries

**DOI:** 10.1186/s40580-024-00417-6

**Published:** 2024-02-26

**Authors:** Zehua Wang, Li Wang, Hao Zhang, Hong Xu, Xiangming He

**Affiliations:** Institute of Nuclear and New Energy Technology, Tsinghua University, Beijing, 100084 China

**Keywords:** Machine learning, Lithium-ion battery material descriptors, Novel material development, Artificial intelligence, Lithium battery development tools

## Abstract

Traditional methods for developing new materials are no longer sufficient to meet the needs of the human energy transition. Machine learning (ML) artificial intelligence (AI) and advancements have caused materials scientists to realize that using AI/ML to accelerate the development of new materials for batteries is a powerful potential tool. Although the use of certain fixed properties of materials as descriptors to act as a bridge between the two separate disciplines of AI and materials chemistry has been widely investigated, many of the descriptors lack universality and accuracy due to a lack of understanding of the mechanisms by which AI/ML operates. Therefore, understanding the underlying operational mechanisms and learning logic of AI/ML has become mandatory for materials scientists to develop more accurate descriptors. To address those challenges, this paper reviews previous work on AI, machine learning and materials descriptors and introduces the basic logic of AI and machine learning to help materials developers understand their operational mechanisms. Meanwhile, the paper also compares the accuracy of different descriptors and their advantages and disadvantages and highlights the great potential value of accurate descriptors in AI/machine learning applications for battery research, as well as the challenges of developing accurate material descriptors.

**Graphical Abstract:**

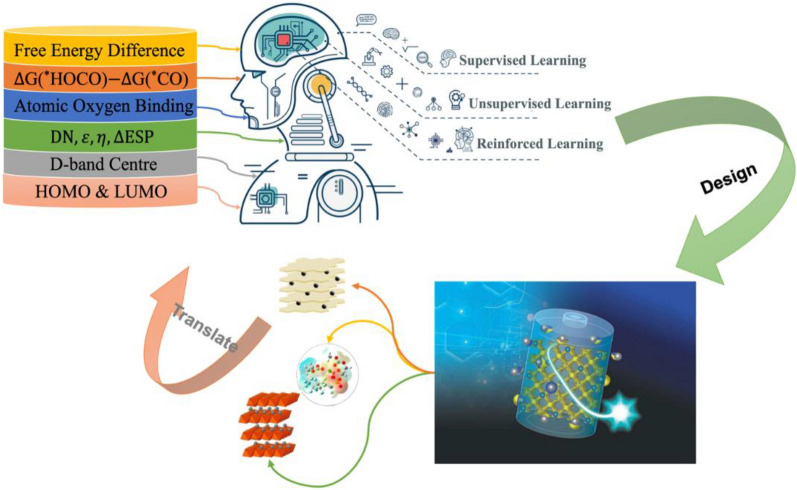

## Introduction

Although LIBs have been very significantly improved since Sony’s success in making the first commercial lithium-ion battery in 1991, with energy densities increasing to more than twice as high, the widespread replacement of fuel vehicles by electric vehicles and the development of energy storage systems require that LIBs are necessarily upgraded in terms of performance, durability, safety, cost, and to reduce their CO_2_ footprint and upgrade their sustainability, which has become a research and development goal for lithium-ion batteries or other next-generation batteries [1]. However, the development of new batteries is a relatively lengthy process, especially as the development of novel energy storage functional materials is influenced by an extremely large number of variables, such as precursor materials, synthesis methods, synthesis environments and morphological characteristics, which make it necessary to deal with large amounts of data for batteries material upgrade, data that sometimes exceed the limits of the arithmetic power of human researchers [2]. Therefore, some relevant international organizations have suggested reducing the experimental workload in battery research by developing new tools and methods to accelerate the development cycle of lithium batteries [3, 4]. The concept of the Materials Genome Initiative was first proposed by then-US President Barack Obama in 2011, to use the computational power of computers to make up for the lack of computational power of human researchers and to overcome the challenges posed by many variables and massive amounts of materials data [5, 6]. While the rise of AI and machine learning has greatly improved data process capability, the lack of accurate material descriptors/genomes has limited the application of AI and ML to materials, which has then become a grand challenge in the Materials Genome Initiative project [5, 7].

Descriptors serve as a bridge between artificial intelligence (AI) and researchers, particularly in the context of physical and chemical material descriptors, an area that has witnessed extensive scrutiny in contemporary research. Nonetheless, a prevailing limitation lies in the specificity of many individual descriptors to systems, thereby impeding the broader utilization of AI methodologies in the examination of functional materials and battery electrochemical systems.

It has proven very difficult to incorporate all the invariants of a molecular system into a single description without compromising its uniqueness or computability. Several geometries map onto the same descriptor, or collide, in a way that some descriptors cannot avoid. Although many solutions to this challenge have been proposed, which are based on general concepts such as density representation, parameter sharing, invariant integration, fingerprinting methods, and NN models that also derive the representation from the data, these solutions in the field of artificial intelligence solutions are unlikely to be applicable or understood by materials researchers [8–11]. Since none of the strategies proposed so far are without trade-offs, the screening and development of an accurate descriptor is currently the major challenge for the use of machine learning in the design and creation of materials for lithium-ion batteries (LIBs). Therefore, the investigation of accurate and low-cost descriptors to build an accurate machine-learning model to help battery researchers and scientists establish the trend of lithium-ion battery discovery through the data-driven method becomes a great challenge.

To address these issues, and to help researchers who are not familiar with artificial intelligence (AI) and machine learning (ML) understand how AI works and how it can affect the development of lithium-ion batteries, the main body of this paper will briefly introduce information about the Materials Genome Initiative, AI, and various machine learning (ML) approaches. Meanwhile, existing descriptors in the lithium-ion battery and functional materials industry field including computational chemistry, electrochemistry, and molecular and ionic dynamics also be reviewed, to help readers develop a systematic knowledge framework of descriptors in the context of lithium-ion batteries. In addition, the underlying logic of descriptors in machine learning will be explored to help the reader gain a deeper understanding and comprehension of the application of machine learning to lithium-ion battery design. Finally, this article will summarise the basic features of descriptors to give the reader a clearer idea of the descriptors used in the field of lithium-ion battery chemistry.

## Materials genome initiative

**Fig. 1:**
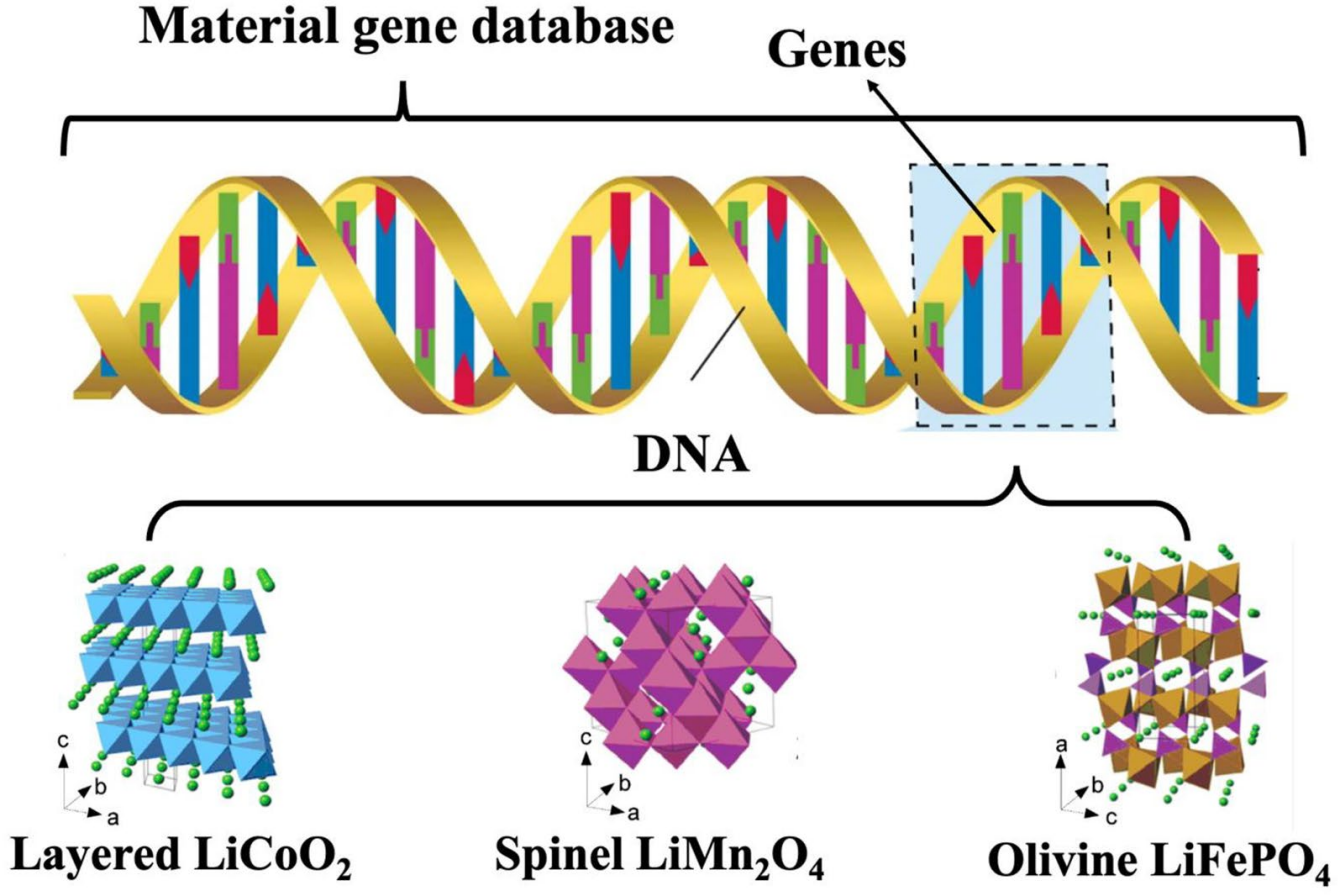
The schematic diagram of the deployment of the Material Genome Initiative [12]

Similar to the Human Genome Project (HGP), the overarching objective of the Material Genome Initiative (MGI) is the establishment of a comprehensive material genes database. This endeavor involves elucidating the intricate correlations between material composition, structure, and properties. The primary aim is to facilitate researchers in discerning the fundamental principles governing material science, thereby enabling the identification of novel high-performance materials, as illustrated in Fig. 1 [6, 7].

The MGI core consists of three modules: high-throughput computation, high-throughput experimental screening and materials database system construction. Materials genome technologies include material conformation characterization, high-throughput computation and screening, neural network technologies, optimization algorithms, machine learning, and high-throughput preparation and characterization technologies. The goal is to understand, design, and compute new materials at the atomic and molecular levels, and to guide the improvement and development of new materials by correlating existing material structures and properties in the database [6, 7]. Where the machine learning part is still a challenge due to the limitations of precise descriptors.

## Artificial intelligence, machine learning and molecular descriptors

### Artificial intelligence and machine learning

The concept of machine learning was first introduced by Arthur Samuel in 1959, but it was not widely accepted due to the restriction of computing power and the limited neural network models [13, 14]. In 1969, Minsky proved that such complex machine-learning models could only solve linear problems, bringing the field of machine learning into the ice age. However, researchers have extended the structure of neural networks to three layers by introducing non-linear activation functions, which greatly broadened the scope of machine learning applications [14]. Meanwhile, Mitchell wrote the classic textbook on machine learning in 1997, which led the academic community to revisit the value of machine learning as a field of study [14]. In the last decade, machine learning has also taken advantage of the explosion in computer science to break through the limitations of three-layer neural networks (the mark of deep learning) and achieve algorithms with the ability to “think” autonomously in the true sense of the word and has a wide range of applications in the modern world [14–16]. Exemplifying the integration of artificial intelligence, contemporary internet search engines, such as Google, employ sophisticated algorithms to analyze and assimilate user search patterns. This analytical capability enables these search engines to dynamically deliver highly relevant search results to individual users. Furthermore, artificial intelligence finds application in various domains, including social networking, where it contributes to personalizing content such as news and short films. It is also instrumental in media content promotion, such as personalized music recommendations, and extends its functionality to image recognition for identifying individuals or objects in pictures. Additionally, artificial intelligence plays a pivotal role in language-related tasks, such as translation services, and contributes to the identification and mitigation of potentially harmful information [15]. In business, AI is used to customise different personalised movie products for different customers, and our mobile phones use AI as personal electronic assistants such as Siri.

Meanwhile, more advanced AI is used in autonomous driving technology, smart grid design and development, and the core of modern robotics [17]. Since AI has several orders of magnitude more computing power than humans, this means that AI can quickly simulate all possible scenarios and determine and give optimal solutions based on the conditions of use [18]. Lithium science follows this trend and to reduce the cost of developing new materials and improve product quality, the lithium industry is investing in artificial intelligence and digitalisation to accelerate its research and development. Meanwhile, academia intends to apply AI and ML as a booster of research in cathode and anode materials, electrolytes (solid/liquid), catalysts and cell structures [19, 20].

**Fig. 2:**
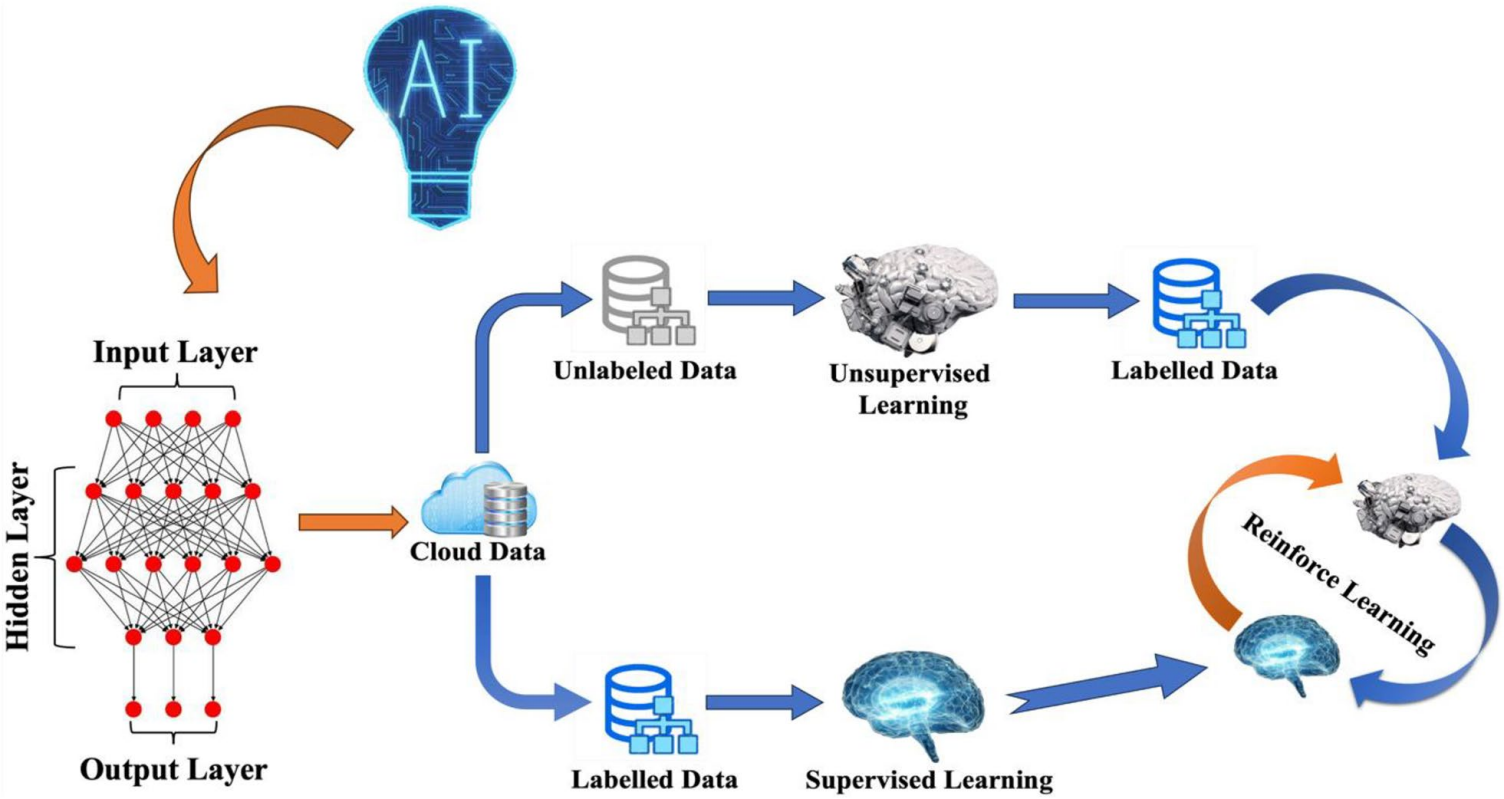
The neural networks and workflow of AI/ML

An ML workflow (Fig. 2) always begins with gathering and preparing the data and encoding the data set into a numerical representation, in which meaningful patterns and regularities can be identified and extracted by the learning algorithm and then translated into the parameters that can be recognized by the computer, and finally to test the model out of sample [21–23].

### Machine learning algorithms and two important elements

Algorithms should be one of the fundamental training elements for machine learning and can be classified as supervised, unsupervised, semi-supervised, and reinforced learning methods [24]. Supervised methods use pre-processed data sets with defined variable inputs and outputs, and in the case of supervised ML, there is a distinction between regression and classification. In unsupervised ML algorithms, this a priori information is missing, and the target is to dig patterns out of the massive dataset. The latter refers to ML techniques that analyze the dataset using classes, whereas the former does it using continuous values. Supervised or unsupervised ML can produce classes used in supervised machine learning. Semi-supervised methods fall somewhere in between, utilizing datasets that contain both labelled and unlabeled data [24]. Reinforcement learning is currently one of the popular methods in machine learning that does not require an explicit dataset as input but provides a virtual environment in which the machine learning algorithms are free to explore and receive the appropriate reward or punishment feedback ultimately maximizing the rewards as in the case of AlphaGo [25]. Deep Learning (DL), also known as “Deep Neural Network Learning Algorithms (DNNs)”, unlike traditional machine learning methods that rely on manually created features, Deep Learning utilizes neural networks to autonomously learn representations from raw data (Table 1), which makes it particularly effective when dealing with complex tasks involving large datasets. The use of multiple layers of hidden neurons (typically no less than 3) combined with highly optimized algorithms and architectures can address the computational power limitations of earlier shallow neural networks (consisting of 1 or 2 layers) and overcome the challenges posed by the inability of manual feature extractors used in traditional machine learning paradigms to efficiently scale large datasets [26, 27].

**Table 1:** Differences between deep learning and traditional ML method [27]

Aspects	Traditional ML	Deep learning
Architecture and representation	Typically relies on feature engineering where human experts manually extract relevant features from the data	Neural networks with multiple hidden layers are involved in the process. They automatically learn a hierarchical representation of the data through the training process, with a relatively weak dependence on feature engineering
Model	Primarily based on supervised learning, unsupervised learning, or semi-supervised learning paradigms	Commonly associated with supervised learning, where the model is trained on labelled large datasets
Data requirements	Often requires a set of handcrafted and high-quality feature data	After training, the model can automatically identify features from raw data, making it adaptable to diverse and complex datasets and capable of handling large quantities of data effectively
Computational capability requirement	Lower	Higher
Interpretability	Models are usually easier to explain and to understand the reasoning behind predictions	Deep neural networks with many layers may be seen as “black boxes” and therefore challenging to explain their decision-making processes
Applicability	Suited for tasks that require explicit feature engineering and prioritise interpretability	Ideal for tasks involving large-scale complex data

Data is another of the fundamental training elements for machine learning, and large amounts of high-quality data are the basis for good performance in machine learning. This data includes both the structure and the properties of materials at all scales. This data can be obtained from a wide range of sources, either from experiments and simulations, published literature or directly from the materials databases, such as ICSD, OQMD, Materials Project, etc. [24].

Material descriptors are defined as descriptive parameters for the material property [28]. Although this concept is very abstract, it is not difficult to understand. Currently, quantities of descriptors have been devised by materials scientists to describe various aspects of material properties. Although material descriptors vary in complexity, they must all satisfy the four characteristics below.

1. Reproducibility means that where the same descriptors are used, the descriptors should be fixed for the same material at any time and place to guarantee the repeatability of machine learning results [28]. For example, to describe the motion of an electron, according to Heisenberg’s Uncertainty principle, simply using the position of the electron as a descriptor does not satisfy the requirement of repeatability, in contrast to the use of wave functions to describe it, which can constitute a reasonable descriptor of the position of the electron [24, 29, 30].
2. Validity means that the descriptor should describe at least one intrinsic property of the material that is strongly relevant to the purpose of the machine learning [28].
3. Distinguishability means that differences between the descriptors of different materials can be recognized by the machine. Theoretically, material descriptors that can be distinguished by humans also should be recognized by machines due to the role of descriptors mainly to bridge the gap between human and machine learning [28].
4. Simplicity means that descriptors cannot be too complex to be learned or understood by machines [28].

## Quantitative structure–activity relationships and molecular descriptors

Quantitative structure–activity relationships (QSARs), which employ mathematical and statistical techniques to explain quantitative patterns of change between the activity or physicochemical properties of a compound and its molecular structure, play a significant role in chemometrics [31, 32]. Since the 1980s, the rapid development of methodology and computational science, especially high-throughput screening, has accumulated a large amount of bioactivity data, making QSAR studies widely used in life sciences, environmental sciences, and other fields [33, 34]. The calculation of molecular descriptors is the basis of QSAR research, and the precise definition and rational use of molecular descriptors are very important in QSAR research [32, 34, 35]. The accurate screen of descriptors is crucial for producing QSAR models with high confidence and validity. Currently, more than 5000 molecular descriptors are available in various software packages [32, 34]. Therefore, the first problem in QSAR research is to select the most relevant ones for the subject under study. The main descriptor selection methods mentioned in the literature are stepwise regression (SR), principal component analysis (PCA), factor analysis (FA) and partial least squares (PLS) [33, 36].

**Fig. 3:**
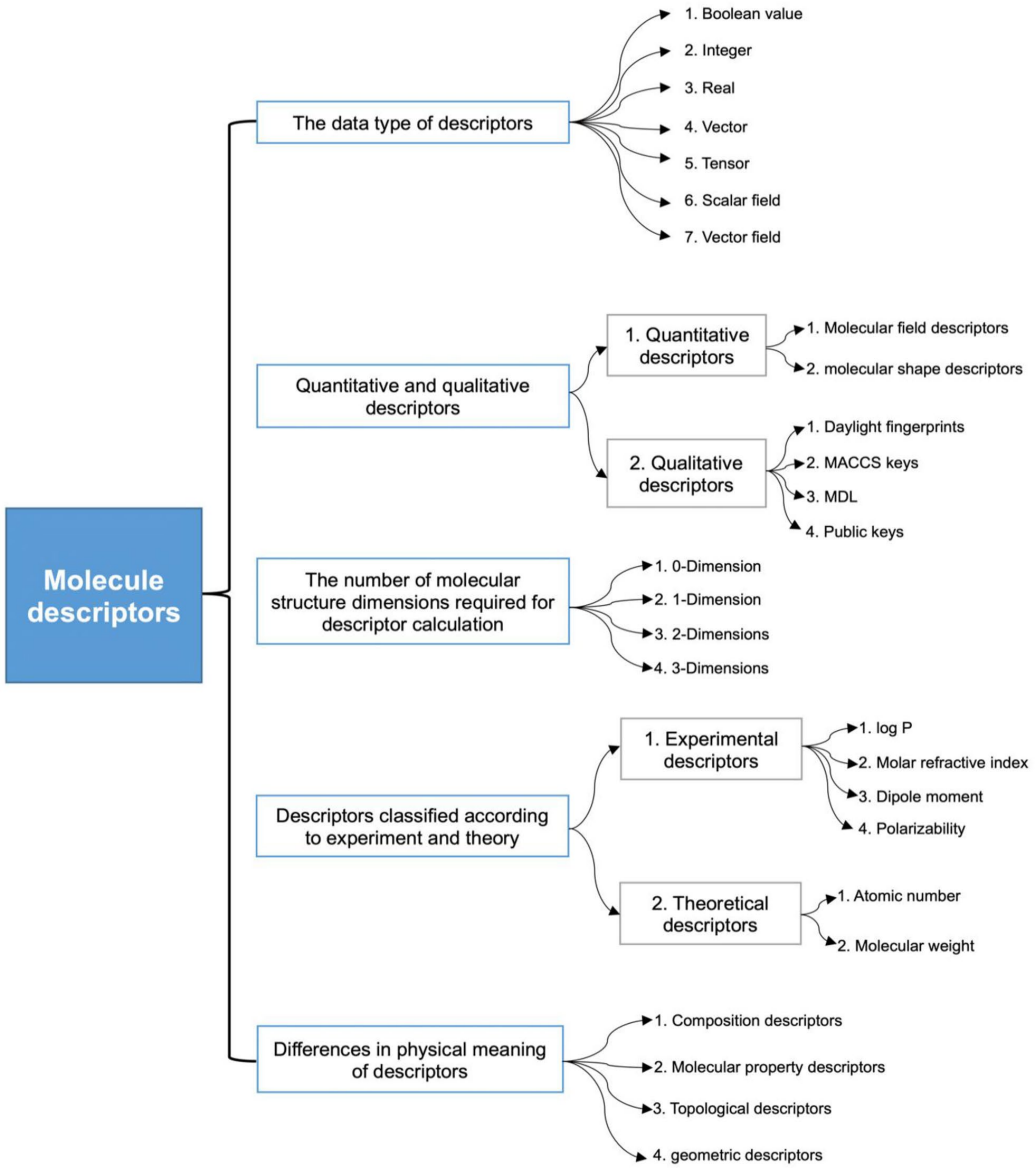
Different types of molecular descriptors

Generally, suitable molecular descriptors have the following characteristics: (1) structural interpretability, (2) an excellent correlation with at least one property, (3) the advantage of distinguishing between isomers, (4) the ability to apply to local structures, (5) outstanding independence, (6) conciseness, (7) not grounded in experimental results, (8) not correlated with other descriptors, (9) can be built effectively, (10) use well-known structural principles, (11) possess the appropriate size dependence, and (12) change as the structure changes. Furthermore, molecular descriptors can roughly be categorized into the types depicted in Fig. 3 [32, 34–36].

Molecular descriptors can be categorized as quantitative and qualitative descriptors [34]. Quantitative descriptors can be based on a variety of theoretical or experimental spectral data, molecular composition (hydrogen bond donor number, chemical bond number), physicochemical characteristics (ester water distribution coefficient), molecular field descriptors, molecular shape descriptors, etc. The structure, characteristics, fragments, or substructures of a molecule are typically represented by a specific code, which is referred to as a qualitative descriptor or molecular fingerprint. Public keys, MACCS keys, Daylight fingerprints, and MDL are examples of molecular fingerprints. Based on the descriptor’s data type, molecular descriptors can be categorized as Boolean (chiral or not), integer (ring number), real (molecular weight), vector (dipole moment), tensor (electron polarization rate), scalar field (electrostatic pattern), vector field (electrostatic potential ladder), and molecular shape descriptor, vector fields (electrostatic potential gradients), and other types [32, 34].

Molecular descriptors can also be classified as one-dimensional, two-dimensional, three-dimensional, etc., depending on the number of dimensions of the molecular structure required for the descriptor calculation. In addition, there are other criteria for classifying molecular descriptors for different computational systems [32, 35]. Take Dragon, a molecular descriptor calculation software, as an example, it can be divided into 20 modules including compositional descriptors, molecular property descriptors, topological descriptors, and geometric descriptors, depending on the physical meaning of the descriptors, each of which represents different chemical information [34].

## Donations of descriptors in Li-cells’ performance improvement and prediction

Typically, the functionality of the cathode, anode, and electrolyte—where the latter also includes lithium salts, organic solvents, and other additives—significantly determines lithium-ion batteries’ performance in a significant way [35]. As mainstream commercial batteries still consist of a solid electrode and a liquid electrolyte, this means that the main processes of lithium-ion migration between the cathode and anode include solid phase diffusion within both electrodes, liquid phase transport in the electrolyte solution and solid–liquid interfacial reactions between the electrolyte and the electrode. Therefore, this review is only concerned with the application of descriptors in solid-state electrodes and liquid electrolytes.

### Electrodes-related descriptors

The mainstream of the widely accepted theoretical models for electrode design currently uses the mean-field pseudo-two-dimensional (P2D) mode [37, 38]. However, the influence of microstructural characteristics on the electrochemical behavior of porous electrodes has been ignored by those design methods, contradicting the axiom that the microstructure of electrodes plays an important role in the performance of electrodes. Recent advances in structural characterization techniques [39], and numerical computational techniques have made it possible to model the microstructure of materials used in lithium-ion electrodes without using volume averaging [40, 41]. For instance, the standard deviation from the state of lithium (SoL), which Lu et al. [42] referred to as the extent of the deviation from SoL as a function of electrode depth, was studied along with the individual SoL of the active layer's particles. They noticed that the standard deviation of the SoL decreases with distance from the diaphragm and converges at a certain depth, known as the solid-state transport (SST) control depth (electrode penetration depth), beyond which the discharge of the particles is severely constrained by liquid-state transport (LST). The results demonstrate that an electrode with a hierarchical particle size distribution can further enhance rate performance without degrading gravimetric energy density using this penetration depth as a descriptor. Moreover, by using SST as a descriptor, the thickness of the electrode sheet can also be designed to optimize the multiplicative performance of the cell without affecting the gravimetric energy density, demonstrating the importance of applying the depth-dependent SoL as a descriptor in optimizing the multiplicative performance of electrode materials when designing layered microstructures for Li-ion batteries [42, 43].

**Fig. 4:**
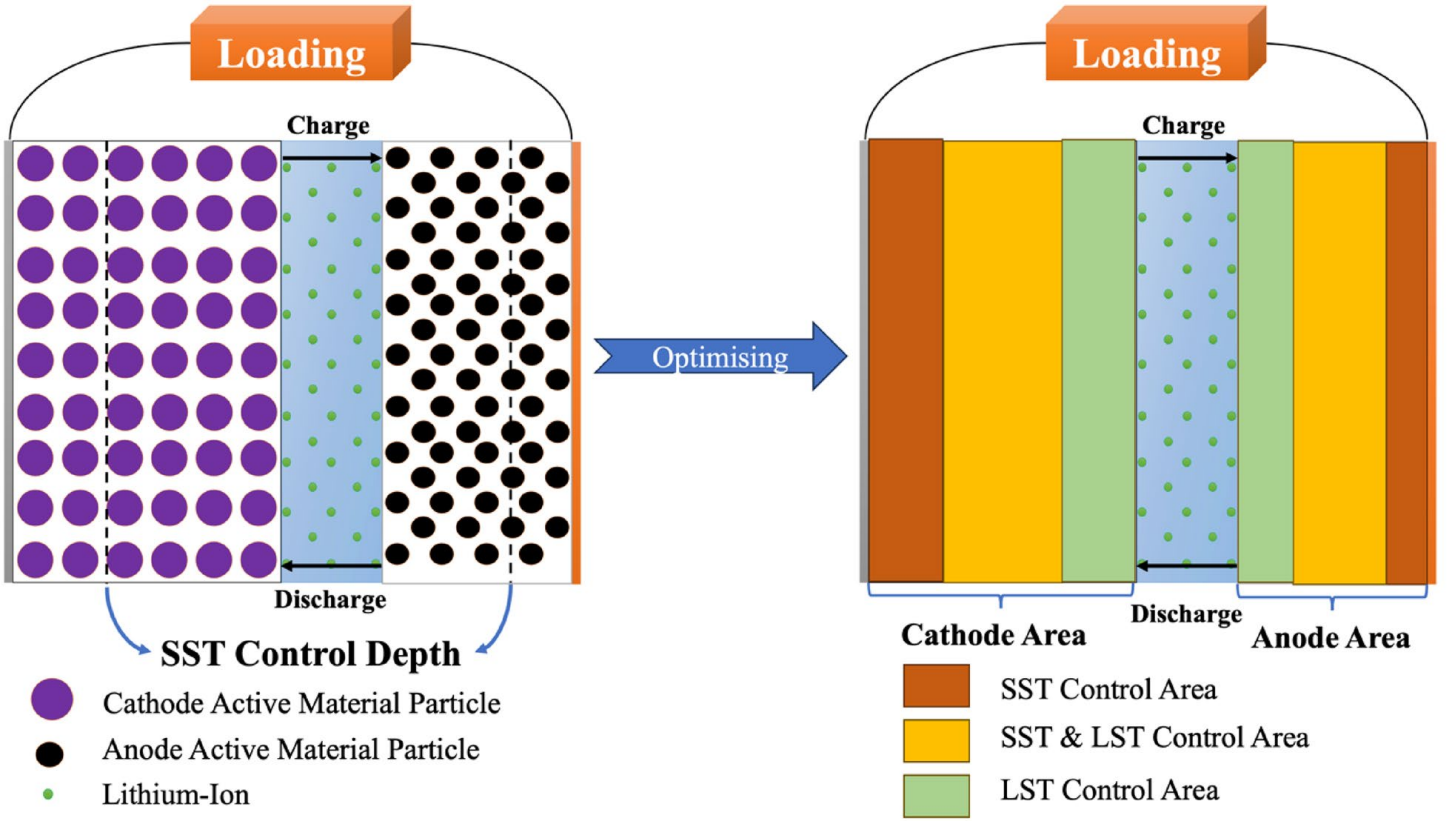
Schematic figure of Li-ion electrode transmission

Furthermore, Shan et al. [43] argues that this modelling of kinetic restrictions in the direction of the electrode depth is too coarse and severely hampers the fine design of the electrode structure. This is because the standard deviation of SoL is shown to vary continuously with depth, rather than decreasing with increasing depth of discharge (DoD), which hinders the accurate identification of the SST control depth. Therefore, they attempted to improve the accuracy of depth-dependent kinetic properties by using the difference in SoL time of individual particles (dSoL) and the SoL span in individual particles (∆SoL) as dual descriptors and dividing the electrodes into SST-controlled, mixed SST-LST-controlled, and LST-controlled zones as Fig. 4 shows, where dSoL stands for the rate at which lithium ions in the particles intercalate and de-intercalate, ∆SoL is the variation in SoL values between the particle’s surface and center, which describes the rate of lithium-ion interfacial charge transfer at each particle’s surface [43].

Electrochemical simulations show that electrodes designed using the dSoL and ∆SoL dual kinetic descriptors have significantly higher capacity at high multiplicity (5C) compared to homogeneous electrodes [43]. This work demonstrates the importance of accurate and stable descriptors when machine learning is applied to optimize lithium battery design solutions, which is consistent with the four properties of descriptors mentioned previously [28, 43].

The integrity, longevity, and safety of lithium-ion batteries have been persistently compromised by the occurrence of fibrous lithium metal dendrite formation on the negative electrode. Such dendritic growth poses a challenge to the reliable performance of the battery over its lifecycle, with instances of extreme usage, such as overcharging, being inevitable. When this happens, the lithium battery anode typically develops a lithium dendrites [44, 45]. When lithium metal precipitates on the negative electrode, it inevitably generates lithium dendrites during subsequent charge and discharge cycles, and they lead to a loss of reversible lithium-ion and active material. As well as puncturing the diaphragm and leading to an internal short circuit accompanied by intense heating, thermal runaway inevitably occurs [44–46]. However, some theoretical calculations have shown that magnesium batteries are more likely to tend to a smooth surface than lithium and sodium batteries during the charging process. [47] The existing theories of dendritic growth are usually not element-specific, which means that they cannot explain the differences between the various metals. Jäckle et al. [48] suggest that the growth mechanism of metal dendrites on the negative surface is closely related to the diffusion process, and a descriptor has been added to predict the rate at which metal dendrites will grow on the negative electrode, the height of the metal self-diffusion barrier. The descriptor’s reliability of using the height of the metal self-diffusion barrier was determined by experimentally comparing the growth of negative dendrites in lithium, sodium, magnesium, and aluminium-ion batteries with different metals. This provides basic design principles for reducing the cost and lead time for the development of anode materials and speeds up the development of dendrite-free electrodes and long-lasting Li-ion batteries.

The development of new cathode materials and the enhancement of the morphological properties of cathode material particles can both improve battery performance due to the cathode material typically determines the energy density of lithium-ion batteries [49]. Nickel-rich cathode materials have received much academic attention due to their high energy density but low-cost advantages [50]. Improving the preparation of its precursors over the process has a significant impact on optimizing the performance of nickel-rich cathode materials [42, 50]. Co-precipitation is currently used as the dominant process to produce nickel-rich cathode material precursors due to its ability to provide secondary particles with a homogeneous composition consisting of highly crystalline primary particles. However, optimization of the preparation process through experimental validation methods is often time-consuming and expensive. Lee et al. [50] suggests a descriptor, which uses the ratio of the reaction quotient (Q) to the equilibrium constant (K) for effective nucleation and crystal growth of metal hydroxides to predict and optimize the production process of nickel cobalt hydroxide (MHP). It was verified that the actual co-precipitated particles had the same and uniform size and shape as predicted, which further evidence of the importance of the role descriptors play in the use of machine learning in the creation of new materials and the improvement of manufacturing procedures.

Moreover, existing cathode materials for lithium-ion batteries are crystals formed by atoms and their coordination environments arranged in the lattice of the basic structural unit and periodicity in a certain combination [51], which is similar to the deoxyribonucleotides in DNA. Therefore, studying the physicochemical properties of cathode materials from an atomic perspective to investigate novel and precise cathode material descriptors, then will lead to a better and deeper understanding of the inherent electrochemical properties of these materials, as well as the rational design of high-performing cathode materials [52].

Marianetti et al. [53] and Maxisch et al. [54] studied the impacts of the coordination environment of different transitional metals in layered transitional metal oxide (Li_x_MO_2_) and polyanionic compound (Li_x_MPO_4_ and Li_x_MSiO_4_) on phase transaction and electronics state on d-orbital changes to predict their conductivities variation. They found that the state of electronics onto the d-band of transitional metal (TM) element in Li_x_TMO_2_ tends to delocalize during the charging process and its crystalline phase structure transforms from a semiconductor phase to a metallic phase with a higher electrical conductivity [53]. By contrast, the state of electronics onto the d-orbital of transitional metal (TM) elements in the polyanionic compound shows the opposite trend, which tends to be more localized and coupled with atomic lattice distortions to form polarons [54]. And, Wei et al. [55] also found that the diffusion of Li ions is closely related to the valence state of TM ions and the size of the lattice through the study of the Li-ion migration in the multi-transition metal oxide. In addition, strong P-O covalence in the PO_4_ tetrahedrons unit serves as joints between the adjunct FeO_6_ planes to create exceptional structural and thermal stability during charge–discharge cycling and prevent oxygen release reactions [56]. By contrast, the irreversible phase transition that occurs during the delithiation process in layered nickel-rich cathode material is caused by the distortion of the unstable NiO_6_ lattice unit, which ultimately results in weak cyclability and reduced capacity retention [57]. Although, the above studies illustrate that the atomic structure units and their coordination method are closely related to several material properties including conductivity, rate capability, and stability, which demonstrates the feasibility of using atomic structure units and their permutations as material descriptors for machine learning to predict material properties, quantifying these structural factors and subsequently integrating them into the realms of machine learning or artificial intelligence poses significant challenges. In response to those challenges, Wang et al. [58] introduced an enhanced iteration of the crystal convolutional neural network (CGCNN) algorithm, denoted as mCGCNN. This advanced deep learning model effectively combines the distinctive features of crystal structures with the physical and chemical properties of materials. It adeptly addresses the data fusion issue inherent in existing generalized models for materials, leading to a notable enhancement in the efficiency and accuracy of predicting gravity capacity for lithium-ion battery materials. The incorporation of scale factors α and β serves to regulate the influence of material crystal structure and numerical data on the model, thereby augmenting the flexibility and adjustability of the overall system, which further demonstrates the efficiency of accurate descriptors paired with appropriate algorithms in predicting the performance of lithium-ion battery materials.

The stability and mechanical strength of the SEI layer also have a significant impact on the cycling stability of lithium-ion batteries, for example, lithium-ion batteries that use silicon-based materials as anodes usually have an extremely short cycle life. In addition to the exaggerated volume changes and expansion stresses of the Si active particles during lithiation and delithiation, the rupture and regeneration of the SEI film during charge/discharge cycles can also deplete the active lithium content significantly, leading to capacity degradation [59–61]. Jiménez et al. [62] and Li et al. [63] tried to solve this issue by coating a robust artificial solid electrolyte interface (ASEI) layer on the silicon particle surface. Nevertheless, the development of an exemplary Solid Electrolyte Interphase (ASEI) layer necessitates the consideration of multiple critical factors, encompassing attributes such as thermal stability, ionic conductivity, and mechanical properties [64]. Applying the mathematical model of the ASEI layer to find key coefficients as descriptors to accelerate the design and development of ASEI film could be an effective method to reduce time and cost. However, most mathematical modelling work on electrodes assumes that the SEI layer is compositionally homogeneous or has a well-defined boundary between the organic and inorganic portions [65, 66]. This contrasts with the actual structure of the SEI film. Therefore, using such less accurate models as descriptors for machine learning can result in outputs that differ significantly from the target [66]. Manoj et al. [64] provide a heterogeneous ASEI model to develop key parameters (SEI film thickness, the thickness of the inorganic–organic interface, elastic deformation and plastic deformation-related parameters etc.) for the ASEI layer. Applying the parameters obtained from the heterogeneous excavated model as machine learning descriptors to accelerate the development of ASEI layers is significantly more efficient than parameters from the homogenized model [67].

### Electrolytes-related descriptors

Lithium nickel manganate (LNMO) [68], which possesses a spinel structure, and lithium cobalt phosphate (LCP) [69], which has an olivine structure, are considered ideal candidates for optimizing the performance of LIBs due to their higher operating voltage and similar capacity to their lower voltage counterparts, LiFePO_4_ (LFP) [69]. One of the requirements for the improvement of high-energy–density lithium-ion batteries is the creation of electrolytes capable of running high-voltage cathodes. However, the development of high-energy–density Li-ion batteries is constrained by the frequently higher development costs and longer development cycles associated with novel electrolytes [70]. The application of ML to battery research is seen as a potent technique for quickening the research process. However, the first hurdle to establishing relevant and accurate AI/ML is frequently the development of appropriate material descriptors, such as precise descriptors of electrolyte oxidative stability. Choosing a reasonable solvent is still a difficult challenge in creating electrolytes [70, 71].

**Fig. 5:**
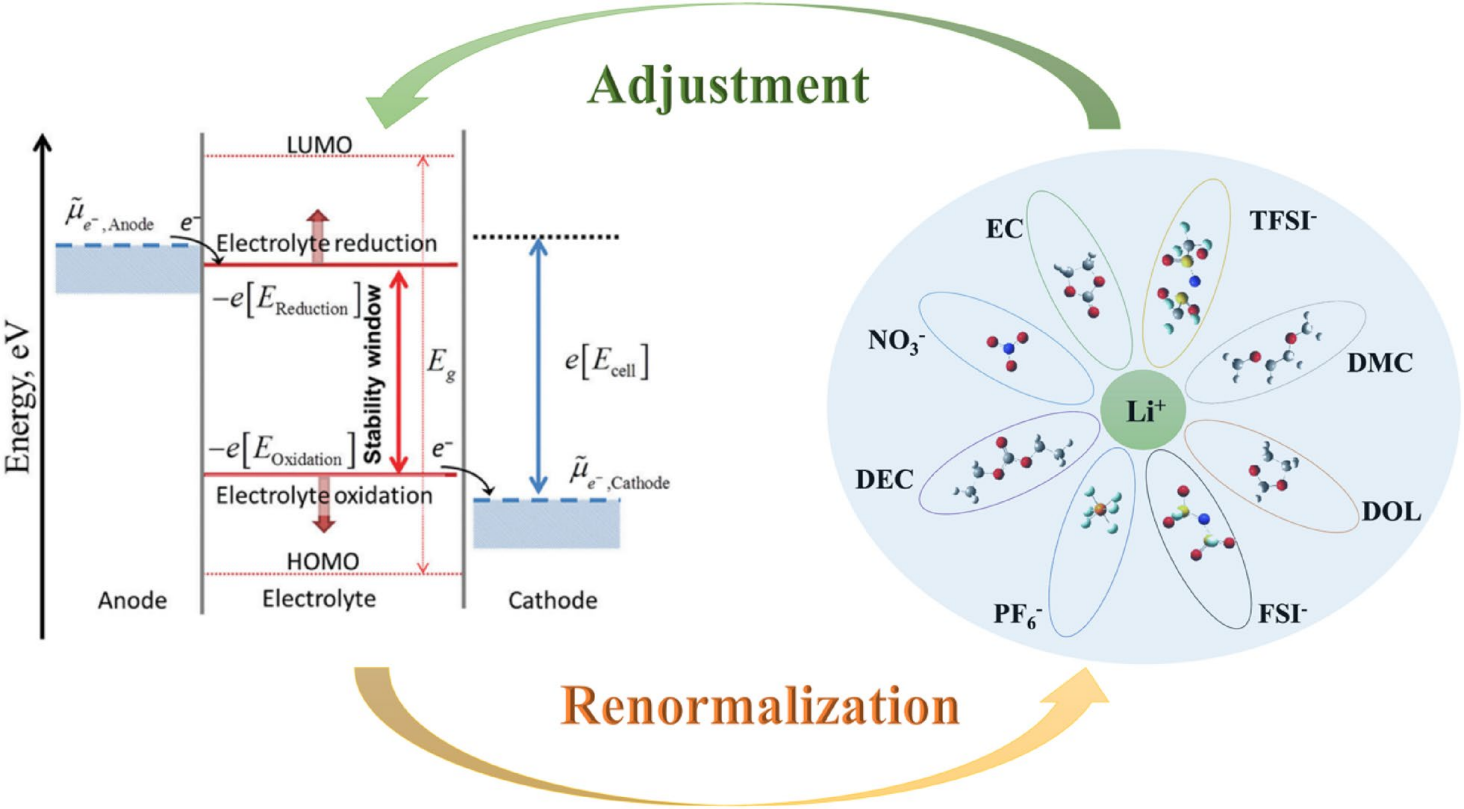
Schematic representation of electrolyte solvation and renormalization

Based on the assumption of a non-interactive environment, the highest occupied molecular orbital (HOMO) level of a solvent molecule is used as a conventional method to define the tendency of an electrolyte to be oxidatively stable [72]. However, the HOMO levels of solvents have been renormalized because, in real electrolyte solutions, solvent molecules are usually encapsulated in their solvation shells as shown in Fig. 5 [70].

**Fig. 6:**
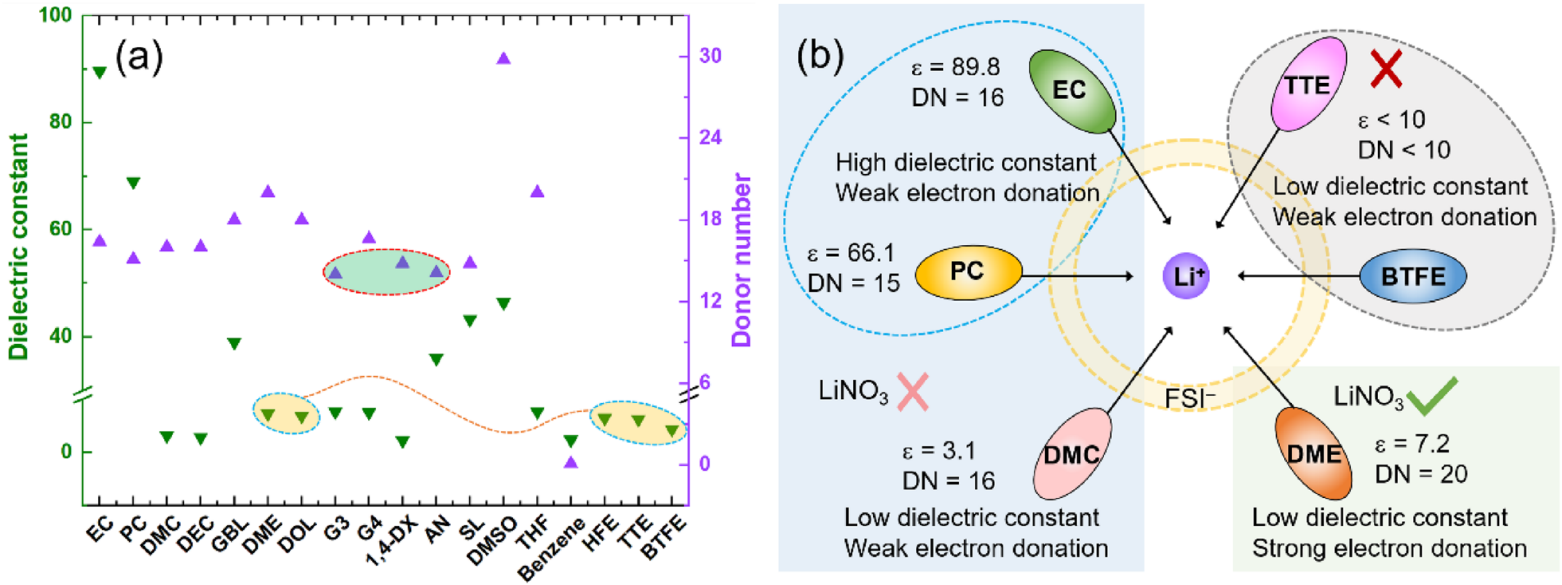
**a** Donor numbers (DN) and dielectric constants (ε) for some solvents used in Li-ion or Li-metal batteries. Reprinted from Ref. [71] with permission. Copyright 2023 Wiley-VCH GmbH; **b** The relationship between the dielectric constant, the number of electron donors, and the solubility of the lithium salt for some common solvents where LiFSI is the primary lithium salt. Reprinted from Ref. [71] with permission. Copyright 2023 Wiley-VCH GmbH

Pande et al. [73] used the number of Gutmann donors and acceptors based on solvent and other components as a simple descriptor of HOMO-level renormalization induced by different electrolyte compositions. This method uses an explicit solvation model and a straightforward generalized gradient approximation (GGA) level DFT calculation to calculate the relationship between the number of Gutmann donors (DN) [74] and the number of donors (AN) [75] of the constituents in the electrolyte and the renormalization of HOMO levels in the solvent. This approach can be used to screen unexplored stable solvents among many known organic compounds for the design of novel high-pressure stable electrolytes and offers a straightforward method for incorporating electrolyte stability in high-throughput computational screening, as opposed to the expensive, experimental data-dependent methods previously employed. Instead, this approach only requires measurements of donor and acceptor numbers. Furthermore, Wu et al. [71]. pointed out that the dielectric constant (ε) and donor number (DN) in electrolyte design lack universality, i.e., they are insufficient to describe a specific solvent’s solubility behaviours. For instance, Fig. 6 demonstrates that although non-soluble hydrofluoric ethers (HFEs) are not soluble in lithium salts, they have a low dielectric constant like that of dimethyl ether. Additionally, lithium bis-(fluoro-sulfonyl)-imide (LiFSI) produces contact ion pairs and is poorly soluble in 1,4-dioxane, even at low concentrations, whereas 1,4-dioxane (1,4-DX) has a greater donor number than acetonitrile (AN) [76].

He et al. [71]. studied the ESP of many solvents using density flooding theory (DFT) and qualitatively analyzed solvent molecules at their highest electrostatic potential (ESPmax) and lowest electrostatic potential (ESPmin). The electrostatic potential (ESP) of the solvent was used as a descriptor for the screening and evaluation of solvents in electrolytes, and it helped to paint a clear image of electrolyte engineering since the ESP can successfully explain how Li+ and the solvent interact in electrolytes [77, 78]. Therefore, the use of ESP as a solvent descriptor can compensate for the lack of universality of dielectric constant and donor number as descriptors, broaden the screening of electrolyte solvents, and is expected to be a tool for implementing effective and accurate AI/ML models, thus accelerating the investigation of advanced and excellent stability electrolytes for outstanding performance lithium batteries. Other material descriptors are briefed in Table 2.

**Table 2:** Summary of material descriptors

Properties	Descriptor	References
The rate of lithium-ion interfacial charge transfer at each particle’s surface	The difference of SoL between the particle’s surface and center	Shan et al. [43]
The ease of dendrite formation on the negative electrode	The metal self-diffusion barrier	Jäckle et al. [47, 48]
Effective nucleation and crystal growth processes of metal hydroxides	The ratio of the reaction quotient to the equilibrium constant (Q/K)	Lee et al. [50]
Material properties	The atomic structure units and their coordination method	Wei et al. [55]
HOMO-level renormalization induced by different electrolyte compositions	the dielectric constant (ε) and donor number (DN)	Pande et al. [73]
To explain how Li+ and the solvent interact in electrolytes	The electrostatic potential (ESP) of the solvent	He et al. [71]
Li bond strength	The chemical shift of Li polysulfides in 7Li NMR spectroscopy	Hou et al. [79]
The CO2RR performance	The free energy difference of $\Delta G$(^*^HOCO) − $\Delta G$(^*^CO)	Chang et al. [80]
The peak of activity	Atomic oxygen binding	Hwang et al. [81]
Catalytic activity	Adsorption energies of Li and LiO_2_	Kim et al. [82]
The electron exchange capacity between two species in the solid–solid interface	The surface acidity	Zhu et al. [83]
The adsorbate–metal interaction	The d-band centre theory	Chen et al. [84]
The oxygen. reduction reaction (ORR)	The rate of ^*^O $\leftrightarrow$ ^*^OH process	Luo and Koper [85]
SEI layer performance	HOMO LUMO Electron affinity (EA) Relatice dipole moment Chemical hardness ($\eta$)	Halls and Tasaki [86] and Wang et al. [87]
Conductivity Rate capability Electronic state on d-orbital Phase transaction	The coordination environment of transitional metal elements	Marianetti et al. [53] and Maxisch et al. [54]
Thermal stability Structure stability	The atomic structure unit stability	Brand et al. [56] Zheng et al. [57]

## Summary and perspectives

This article reviews the basic concepts of AI/ML, algorithms, and relevant descriptors in the context of lithium battery materials. It also discusses the importance of appropriate and accurate descriptors in the application of ML to accelerate the development process of novel battery materials.

**Fig. 7:**
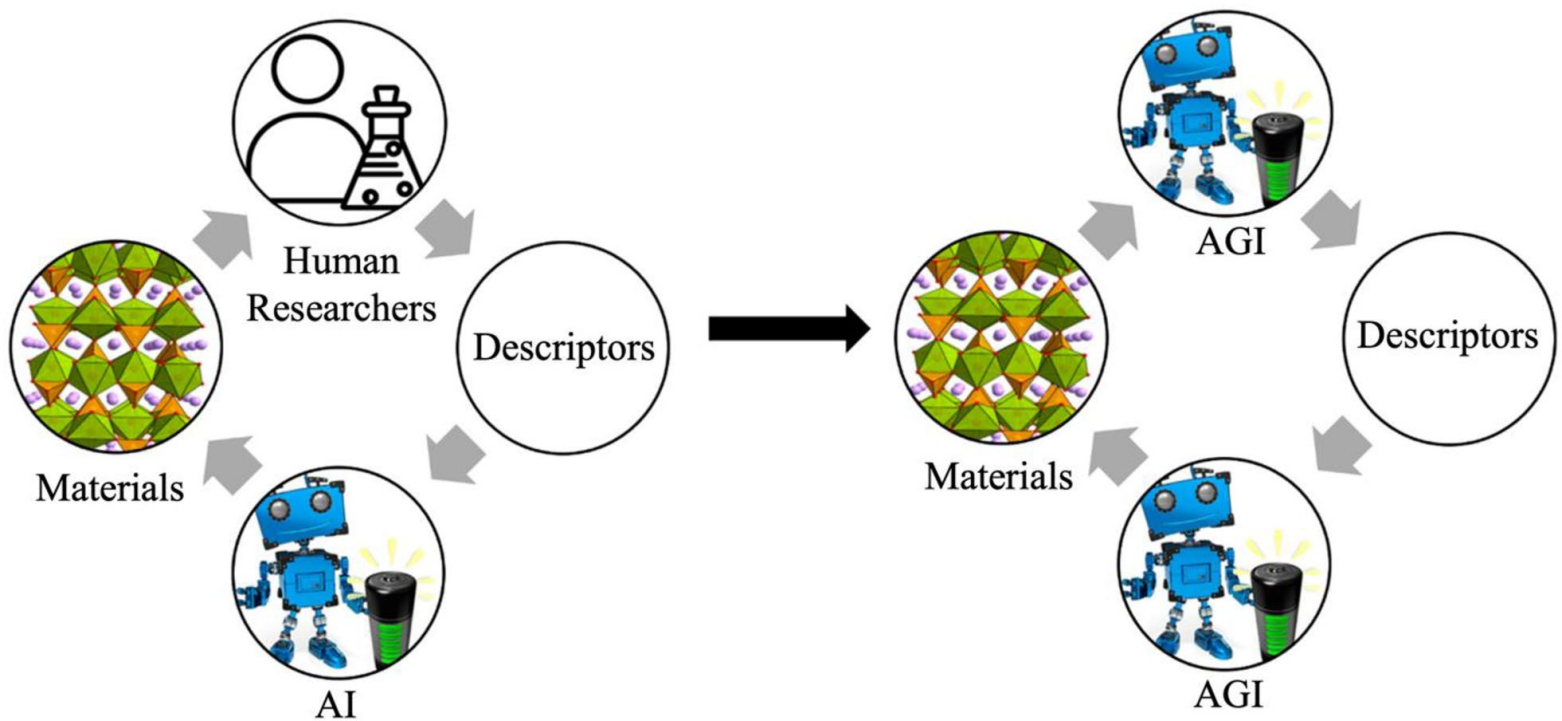
Evolution of the approach to the development of new materials through artificial intelligence

In essence, the progression of material descriptors is intricately tied to the evolution of Li-ion battery technology. This involves parameterizing specific intrinsic characteristics of materials, such as electrostatic potential, donor number, dielectric constant, and atomic structure units. Similar to the intricate code embedded in human DNA, material descriptors are poised to effectively articulate, predict, and facilitate the deliberate manipulation of corresponding materials at the foundational level of atomic structure. Simultaneously, for these descriptors to be impactful, they must exhibit qualities of reproducibility, simplicity, efficiency, and accuracy. Given the iterative nature of machine learning, the accuracy of material descriptors demonstrates an exponentially positive correlation with operational efficiency. Consequently, more precise descriptors play a vital role in expediting machine learning to accelerate the development of novel materials. Moreover, as artificial intelligence continues to progress and in-depth research on relevant material descriptors expands, the widespread adoption of AI/ML in material development and design will inevitably increase [88]. Notably, the advent of Artificial General Intelligence (AGI) marks a significant stride in the next generation of AI, empowering machines with comprehension, learning, and knowledge application across a broad spectrum of tasks at a level comparable to human capabilities. AGI’s general cognitive abilities, akin to diverse human skills, imply adaptability to new and unfamiliar tasks without explicit programming. [89–91] With the anticipated arrival of AGI, material descriptors are projected to acquire self-scaling capabilities, ushering in a new era of fully automated advancements in Li-ion battery technology and other material developments (Fig. 7).

## Data Availability

The data required in the article can be obtained from the author.
